# Combining community-based system dynamics and design thinking to inform public health intervention: a case study optimizing community-clinical linkage design in Brooklyn, NY

**DOI:** 10.3389/fpubh.2025.1585633

**Published:** 2025-06-04

**Authors:** K. Toney, E. Ballard, J. Duch, C. Zuniga, R. Gore, A. Castaneda, I. Dapkins, B. Roy

**Affiliations:** ^1^Tributary Design, Boston, MA, United States; ^2^Brown School at Washington University in St. Louis, St. Louis, MO, United States; ^3^Department of Population Health, NYU Grossman School of Medicine, New York, NY, United States; ^4^ParentChild+, New York, NY, United States; ^5^Family Health Centers at NYU Langone, Brooklyn, NY, United States; ^6^NYU Langone Hospital–Brooklyn, Brooklyn, NY, United States; ^7^Department of Medicine, NYU Grossman School of Medicine, New York, NY, United States

**Keywords:** systems thinking, system dynamics, design thinking, participatory research, social determinants of health, complexity

## Abstract

The underlying drivers and outcomes of social determinants of health are dynamically complex, making it difficult to design effective responses. This complexity has inspired a growing number of calls to move beyond mechanistic thinking and use systems science to engage directly with complexity and highlight opportunities for methodological innovation to enhance translation of insight into real world action. This case study describes a methodological innovation combining community-based system dynamics and design thinking to understand multi-level complexity of a public health challenge: optimizing the design of a community-clinical linkage in Brooklyn, New York. In-depth description of the case illustrates methods integration and resulting insights and recommendations. Results from the case demonstrate that integrating methods generates insight at multiple levels, including connecting holistic system understanding to individual experiences of system structure and operationalizing and translating insights into action. Combining community-based system dynamics and design thinking holds value for intervention planning, strategic implementation, and sustaining change.

## 1 Background

Social determinants of health (SDoH) have significant negative health effects among low-income, racial/ethnic minority, and immigrant populations (1). Interconnected causes of these poor health outcomes include factors related to poorer built environments, access to healthy foods, access to safe spaces for activity, access to medical care, and social and psychosocial supports. SDoH transcend specific disease states and negatively impact outcomes across conditions, with up to 70% of premature deaths in the US attributed to these factors (2).

Despite their significant negative impacts on health outcomes, addressing SDoH has traditionally largely fallen outside the domain of hospitals and clinics (2–4). Recent efforts emerged to include assessing and addressing SDoH as part of innovative healthcare models (e.g., accountable health communities, patient-centered medical homes) (5, 6). Further, federally qualified health centers were established to serve the community’s social and health needs (7). While evidence from these models demonstrate that routinely addressing patients’ SDoH (e.g., food insecurity, housing stability) during clinical visits can improve health outcomes (8–10), success requires partnership with community-based organizations or other social service-providing entities.

### 1.1 Community-clinic linkage

Community-clinic linkage (CCL) models reflect such collaborative efforts to comprehensively address health and social needs. CCLs are coordinated partnerships among healthcare providers, community-based organizations (CBOs) that provide social services, and public health agencies that aim to improve patients’ access to preventive care, chronic disease care, and social services (11, 12). CCLs include components such as referral systems to CBOs and community health worker (CHW) navigation, and these models are proliferating nationally, across the US. A CCL creates a continuum of care that moves beyond a patient’s clinical needs to include broader social services like housing or transportation. Studies document that establishing a CCL has the potential to improve health outcomes such as: hypertension and diabetes (13), BMI and weight loss (14–16) alcohol and tobacco use (14) physical activity (14, 17) and emotional well-being (18). Studies drawing on more established practices, such as social prescribing in the UK, also highlight potential barriers to implementation such as resource constraints, training limitations, and accessibility challenges; and facilitators such as the importance of relationships, collaboration, and integration (19).

CCL models are evolving across large health systems in the U. S. to connect, communicate, and coordinate across healthcare, public health, and CBOs to address barriers to achieving optimal health faced by vulnerable populations. An integrated, functioning CCL is complex with infrastructure acting at multiple levels of the socio-ecological model and includes networked and representative partnerships, relational engagement with patients/clients themselves, engaged data, and multilevel leadership (20, 21). These CCL models are inherently dynamic and interconnected, including activities to develop and sustain the CCL within community-based organizations and health systems, and interactions among them. Among organizations, members of the CCLs with potentially competing priorities or disparate incentives must work together to support community members using a finite set of resources. Within organizations, leaders and staff must introduce new workflows to communicate with external organizations and with patients and clients, collect information from them, integrate this information internally and/or share back information externally. These multi-level complexities make CCL design an ideal illustrative example for the application of integrated systems science and design thinking.

Within organizations, implementing evidence-based CCL strategies such as digital screening and referral platforms linking healthcare and CBOs to facilitate communication among organizations or engaging CHWs to coordinate services to meet social needs may not be fully adopted due to competing priorities, time constraints, and mismatched incentives (22). Realizing the full potential of these services requires active, tailored changes in care processes that are responsive to the local environment and population. At the community-level (23), social service agencies may not have sufficient capacity to meet referred patients’ needs, and studies show only one-third of social needs referrals are completed (24, 25). Further, capacity of social service agencies vary greatly by community and by type of social need (e.g., food vs. housing insecurity). As such, community-wide coordination of service capacity among constituent CCL organizations is needed for efficient resource utilization and supply–demand matching.

### 1.2 Methods to understand multi-level complexity

Recent discourse in public health has argued for a new model of evidence that engages with the complexity of systems rather than controlling for it in order to improve outcomes (26). This call builds on arguments that position interventions as events acting on systems that evolve and adapt (27), and acknowledges the limitations of traditional epidemiological methods to address the dynamic nature of systems and existence of feedback mechanisms (28). Systems science methods provide a suite of tools and approaches to engage with the structure and complexity of systems as the subject of inquiry (29). A growing number of applications of systems science methods to public health issues and connection to social services highlight both growing interest in this work, as well as opportunities for methodological innovation to enhance translation of insight to real world action (30–35).

System dynamics is a systems science method that uses qualitative diagramming and mathematical modeling to represent and understand the implications of accumulation, delay, and feedback on the behavior of systems (36–38). System dynamics modeling has been applied to a wide range of health and healthcare related domains, with an emphasis on healthcare operations and dynamics of noncommunicable disease prevention and response initiatives (39). Unique features of system dynamics make it particularly useful for translation of insight to action in health system transformation– the operational perspective of system dynamics explicitly represents the material flows of people and resources and the information structure through which actors make decisions to manage systems, allowing actors to engage with model conceptualization and critique (40). Visual diagramming conventions support participatory modeling and group model building, so that community and healthcare system partners can jointly conceptualize models, generate analysis and insight, and build buy-in for action (41). Finally, the pairing of visual conventions with an underlying system of differential equations allows the use of system dynamics simulation to build confidence in exploratory models and test hypothesized mechanisms of change (42).

Community-based system dynamics is an approach within the broader field of system dynamics that builds on the rich tradition of participatory modeling with an explicit emphasis on building community and organizational capabilities for lasting systems change (43). Community-based system dynamics uses the methods of group model building, or structured participatory activities to engage key stakeholders in modeling, and system dynamics simulation. The approach differentiates itself from other traditions of system dynamics methods through the utilization of collaborative planning, facilitation, and analysis by a core team that comprises system dynamics modelers, those with substantive expertise in the topic of interest, and individuals with deep community relationships in order to ensure that the insights from the process and potential impacts of modeling work last beyond the end of a specific modeling project. Community-based system dynamics as an approach has been applied in multiple public health domains (44).

While advances in the use of systems science methods such as system dynamics show promise, there have been calls for integrating complementary methods to advance public health. Systems science can provide insight into complexity and elicit diverse perspectives of public health problems, but systems science methods such as system dynamics provide little structured process to support translation of insight into tangible solutions and steps for action. Huang et al. (45) call for integrating systems science with design thinking to advance public health innovation. Design thinking is a problem-solving approach that engages end-users in the process of developing solutions. Design thinking includes activities such as end-user interviews, direct observation and has been used to support problem solving in healthcare contexts, enhancing patient experience, and development of public health solutions (46–49). There is an opportunity to integrate methods to engage stakeholders so that they can collectively understand multi-level complexity, to elicit diverse perspectives involved, to understand patient experience and clinical workflow within the context of a system, to test assumptions about potential interventions, and to mobilize insight into action. There is limited research describing the application of complementary methods for advancing public health.

This paper presents a case study demonstrating methodological innovation of combining community-based system dynamics and design thinking to understand multi-level complexity of a public health challenge: optimizing the design of a CCL in Sunset Park, a diverse, multi-ethnic, immigrant community in Brooklyn, New York.

## 2 Case presentation

### 2.1 Setting

The Sunset Park community of Brooklyn, NY is a residential, industrial and commercial neighborhood that has been a long-time home to new immigrants. Sunset Park has a population of 146,000, 40% of whom identify as Asian, 39% Latine, and 16% white. Nearly half were born outside the United States, more than 75% speak a language other than English, and over half speak English less than very well. Almost 40% of residents have less than a high school degree, with 22% living in poverty. Sunset Park has the highest density of Medicaid recipients in the country.

The Family Health Centers at NYU Langone is a community-based health network that provides high-quality primary and preventive care to adults and children regardless of their ability to pay or health insurance status. Founded in 1967, FHC has been dedicated to reducing barriers and improving access to health, education, and social services for ethnically diverse, medically underserved neighborhoods in NYC, primarily in Brooklyn. A Federally Qualified Health Center, the FHC has evolved into one of the largest and most comprehensive community health center networks in New York, as well as in the country. The FHC serves approximately 110,000 patients across nine multi-disciplinary primary care sites that support more than 80% of all Medicaid recipients in the area. The FHC also has a comprehensive array of family support and social service programs that are responsive to evolving family and community issues and affirm each family’s cultural, ethnic, and linguistic identities. These services fall under FHC’s Department of Community Services, which also include programs providing early childhood services, youth development, adult and family education, food insecurity, family literacy, parenting and family support, and older adult programming.

In 2023, NYU Langone-Brooklyn Hospital launched an initiative to create a CCL in partnership with the FHCs and 10 partner CBOs located in Sunset Park. These CBOs provide a wide range of advocacy and social supports for the ethnically diverse Sunset Park, including older adult services, medically-tailored meal delivery, benefits enrollment, housing, care coordination, immigrant services, legal services, childcare and after-school programs, English classes, and tax preparation, among others. Many CBOs engaged in the CCL, particularly those serving the Mexican and Chinese communities in Sunset Park, demonstrate deep cultural and linguistic competence in their service delivery. These organizations provide critical mental health supports, including culturally attuned workshops, community groups, and direct services that align with the cultural norms and communication styles of immigrant populations.

Together, building on the longstanding relationship the hospital and the FHC enjoyed with CBOs, they planned to design and implement a CCL model to coordinate across health and social care systems to address the unique health and social needs of local community members, with an overarching goal to equitably improve health and well-being in the community. To achieve this goal, the planned CCL incorporated technology solutions to support digital assessments and electronic referrals for myriad health and social care needs across these organizations, facilitated by CHW navigation. The launch of the initiative was highly timely, as New York state received approval for an 1115 Medicaid Demonstration Waiver amendment in 2024, which supports health systems and CBOs to screen all Medicaid recipients for health-related social needs and place referrals to meet any identified needs using a digital referral system (50). While FHC had been screening for SDoH in a subset of their practices, they noted low rates of screener completion.

The contexts of CCLs are dynamically complex, featuring interconnections between health systems, community organizations, and communities that may change over time; and involving multiple stakeholders to develop and maintain them; as well as incorporating workflows that need to serve diverse, high-need populations and that cross organizations – making CCL design an ideal application for community-based system dynamics and design thinking.

### 2.2 Methods

The project team used community-based system dynamics and design thinking methods to optimize the design of the CCL in the Sunset Park community. All activities were completed across three phases over a 6 month period, led by a core project team of 14 people, including program staff, two external consultants, and representatives from the FHC and the community.

The objective of Phase 1 was to explore existing linkages between community and clinical settings from diverse perspectives. Group model building, informed by a community-based system dynamics approach, was used to convene health care and social service providers to elicit their perspective of the complexity of the CCL and existing approaches to identifying and addressing SDoH. Phase 2 focused on one practice within the FHC to investigate SDoH screening and referral pathways. Design thinking was used to elicit how individuals experience the system structures identified through group model building. The objective of Phase 3 was to test the potential impact of recommendations to optimize the CCL design. System dynamics simulation modeling was used to simulate patient flows and capacity dynamics within and across health and social care organizations to further operationalize and test assumptions of emerging recommendations for action.

Underpinning all three phases was a deep collaborative approach, informed by principles of community-based system dynamics. The core project team invested in close collaboration to evolve the methods based on the priorities and insights of people living and working in the system. The core project team participated in iterative adaptation to approaches used, participatory sense-making and action-planning, and capability development to enable future process replication. See Table 1 for an overview of methods and approaches used across phases.

**Table 1:** Overview of methods including description of activities and who was engaged.

Activity	Description	People engaged
Core project team engagement: adapt and tailor the planned methods throughout all project phases and build capabilities for future replication		
Experiential learning workshops	Train 14 people in community-based system dynamics and design thinking	Core project team
Planning	Weekly calls to tailor methods, prepare for co-facilitation, and synthesize outputs	Core project team
Status updates	Biweekly status calls to update leadership on project status to ensure ongoing buy-in for process and share emerging insights	Project leadership & health system leadership
Phase 1. Group model building: develop holistic understanding of the community-wide CCL system		
Structure elicitation sessions	Two parallel sessions with 30–40 participants each to map the system of addressing health and social needs	Health system & CBO staff
Offline synthesis	Working calls to synthesize outputs from the structured elicitation sessions into a provisional causal loop diagram	Core project team
Structure refinement & intervention elicitation sessions	Two parallel sessions with 30–40 participants each to refine the synthesized causal loop diagram and elicit new and existing interventions for system transformation	Health system & CBO staff
Phase 2. Design thinking: investigate individual experiences of the CCL system structure identified in Phase 1		
Interviews	Interviews with ~25 people to understand individual experiences of SDoH screening and referral in one clinic	Patients, health system & CBO staff
Direct observations	Public observations in one clinic and one social service center to understand impact of physical space	Patients & health system staff
Walkthroughs	Virtual walkthroughs of practice workflows and IT platforms to understand the role of supporting tools in the SDoH screening and referral process	Health system staff
Site visits	In-person visits at 5 CBO partner sites to discuss processes for receiving referrals and service delivery strengths and challenges	CBO staff
Phase 3. System dynamics simulation: test potential impacts of intervention scenarios based on emerging insights and recommendations from Phases 1 and 2		
Initial model development	Build initial simulation model leveraging generic healthcare system capacity structures from the literature and key feedback identified during group model building workshops	Core project team
Consultative model development & calibration	5 working calls with a subset of the core project team, the FHC data teams, quality improvement teams, and social service providers to refine model and inform parameter estimates and model calibration	Core project team, FHC data & quality improvement, CBO staff
Interface development	Develop interactive interface to enable non-modelers to engage directly with the simulation model and explore impacts of interventions on model simulation runs	Core project team

#### 2.2.1 Core project team activities

To align with the explicit emphasis of community-based system dynamics on building community and organizational capabilities for lasting systems change, we engaged the core project team in a series of two-day experiential workshops (four total) to build their capabilities in community-based system dynamics and design thinking approaches. Each experiential workshop introduced the methodological foundations to be used in the phase and gave the core project team opportunities to participate in experiential learning and refine the planned approach. During the workshops, the core project team also collaboratively synthesized outputs of the previous phase and critically reflected on methods used in the previous phase to identify opportunities to improve subsequent activities and inform future process replication. Throughout the project, the core project team participated in weekly planning calls to tailor the approach and select members of the team co-facilitated activities and/or supported synthesis. Additional members outside of the core project team supported more in-depth activities such as participating in simulation model development in Phase 3.

Project leadership and external consultants held biweekly status meetings with health system leadership to ensure ongoing buy-in for the process and emerging insights. At the end of the project period, the core project team facilitated a retreat to share results with over 50 health and social service providers.

#### 2.2.2 Phase 1: group model building

The first phase of the project was a series of participatory group model building workshops to develop a holistic vision of the CCL system in Sunset Park as it currently existed, and to identify both challenges and opportunities for transformation. The workshop was designed to fulfill a set of objectives: (1) To convene and engage varied perspectives including healthcare providers, hospitalists, CBOs, and community advocates; (2) To expand participants’ current conceptual boundaries of the system for addressing social needs within and beyond the healthcare system; (3) to identify opportunities for action to enhance the design and performance of the CCL system. Two parallel two-session workshops were hosted at local community spaces in the Sunset Park neighborhood comprising between 30 and 40 participants per session. Each session was 3 hours long and structured using a variety of group model building scripts tailored by the core project team (51, 52). Participants with varied perspectives were invited to attend either workshop based on their availability and were encouraged to attend both sessions. The first pair of workshop sessions focused on mapping the system of social care over time and included activities for framing the challenge around identifying and addressing social needs, eliciting structural factors influencing the ability of the system to address social needs, and mapping the interconnections between these factors through causal loop diagrams (53). Between sessions, the core team rapidly synthesized the participant-generated diagrams to generate a provisional causal loop diagram. In the second pair of workshop sessions, the team presented a synthesized causal loop diagram for review and live editing, then used this refined map to crowd-source existing efforts already working in this system to create change, and to elicit new ideas for system transformation. The workshops identified a range of intervention strategies that would be refined and tested in subsequent phases of work. The core team continued to refine the causal loop diagram in subsequent phases of work.

#### 2.2.3 Phase 2: design thinking

Based on the system insights and emerging opportunity areas identified using group model building, the core project team selected an FHC practice for deeper investigation of individual experiences of system structure, specifically to identify opportunities to enhance the SDoH screening and referral experience. The practice was selected because it had an existing workflow for SDoH screening and referral. Design thinking activities were used to engage end-users, broadly defined as patients and caregivers, healthcare clinicians and administrators, social service providers within the health system, and partner CBO staff. While patients and caregivers have an essential perspective of the patient experience, engagement was not limited to patients and caregivers since the primary users of the potential solution (i.e., technology solutions, EHR integrations, workflows, etc) were likely to be healthcare or social service stakeholders. Design thinking activities included end-user interviews, direct observation of public areas in the practice and internal social services, walkthroughs of practice workflows and other processes, and partner CBO site visits. Findings were synthesized into process maps and personas. Process maps described key steps in the screening and referral process, stakeholder interactions, supporting tools, and strengths and challenges at each step. Personas are fictional characters developed from design activities to represent typical users or key stakeholders of products or services. The core team participated in multiple rounds of synthesis to refine the final process maps and personas and support identifying opportunities for change to improve the experience.

#### 2.2.4 Phase 3: system dynamics simulation

Emerging insights and recommendations from group model building and design thinking activities were leveraged to develop a system dynamics simulation model to test potential impacts of intervention scenarios in a virtual environment. The purpose of the system dynamics simulation was to operationalize assumptions about the ways patients flow through the system and explore how resource dynamics and delays may influence patient throughput and capacity strain.

The model was developed by a subset of the core project team in close consultation with members of the FHC data teams, quality improvement teams, and social service providers through a series of consultative calls and model development work. Initial conceptualization leveraged generic healthcare system capacity structures from the literature (54) and was grounded in the core feedback structures and dynamics identified through group model building workshops in Phase 1. The core project team also developed an interactive interface to enable non-modelers to engage directly with the simulation model and explore impacts of interventions on model simulation runs. All modeling was completed in Stella Architect (Version 3.4.3), a system dynamics modeling software (55). In line with a collaborative community-based system dynamics approach, multiple stakeholders were involved in model development. The core project team and representatives from other FHC teams such as quality improvement and data reporting met to refine model boundaries, inform parameter estimates, and develop trend data to inform model calibration.

### 2.3 Case results

The project resulted in a series of outputs representing structural barriers and facilitators to identifying and addressing SDoH, insights into how individuals experience the process and broader CCL, and key points of intervention to improve identifying and addressing SDoH. Insights from each phase culminated in a series of recommendations to strengthen the CCL and improve the SDoH screening and referral process in the FHC at Sunset Park. The following section is organized by a description of the outputs and illustrative insights from each phase, and closes with the resulting recommendations.

#### 2.3.1 Phase 1 outputs

**Figure 1:**
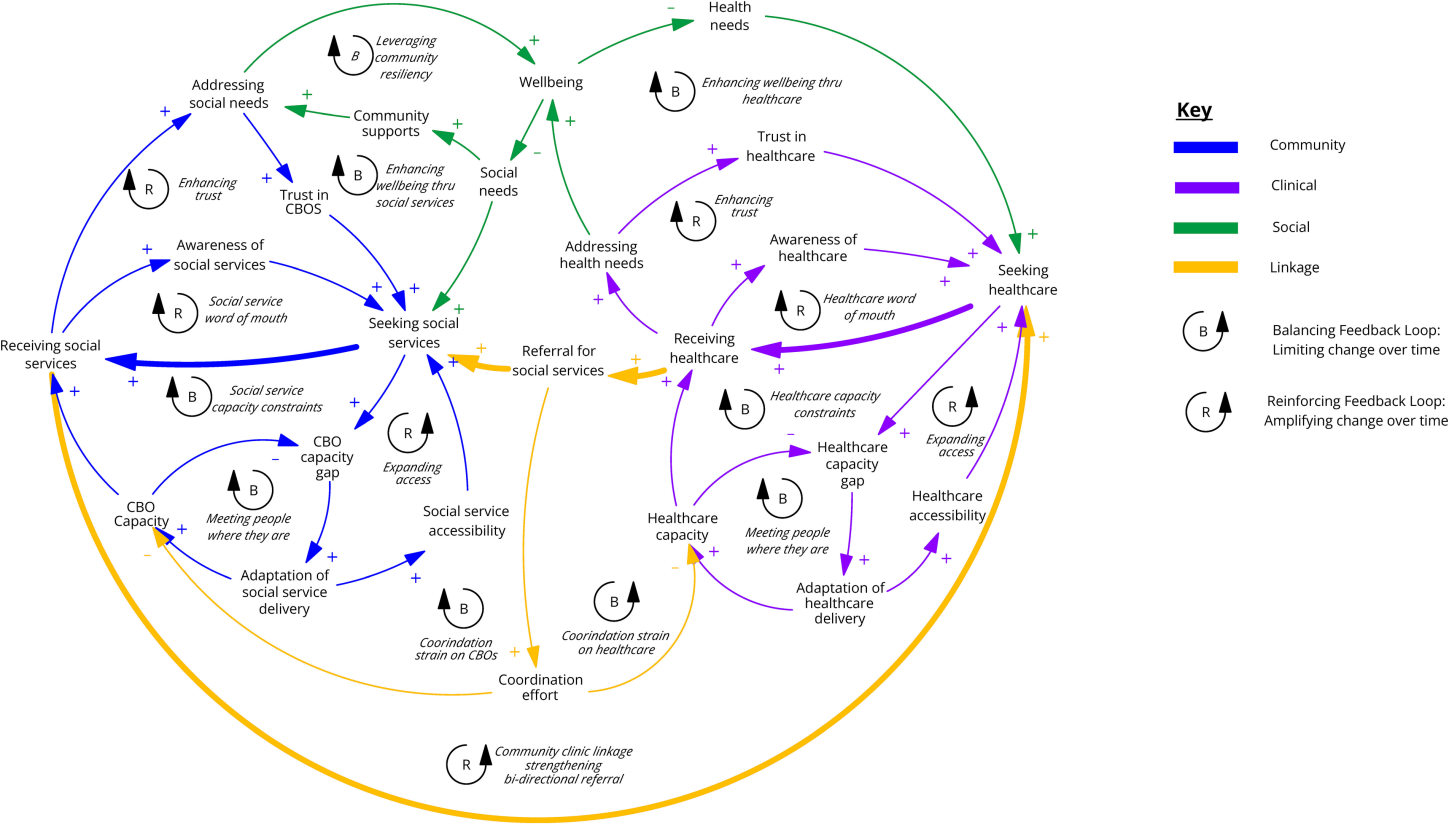
Causal loop diagram where arrows with “+” indicate variables are changing in the same direction (an increase in the cause variable leads to an increase in the receiving variable or a decrease leads to a decrease). Arrows with “−” (negative polarity) indicate variables are changing in opposite directions (an increase in the cause variable leads to a decrease in the receiving variable or a decrease leads to an increase). Note, in this diagram the bolded arrows highlight the key feedback loop signifying community and clinical linkage.

Group model building activities in Phase 1 produced (1) factors related to addressing social needs and how those factors have changed over time; (2) a synthesized causal loop diagram visualizing community and clinical connections to address social needs; and (3) brainstormed opportunities for system transformation. Figure 1 shows a synthesized causal loop diagram demonstrating the interconnections between structural factors influencing the CCL and addressing SDoH.

#### 2.3.2 Phase 1 illustrative insights

Preliminary insights from this phase highlighted the parallel dynamics of care-seeking, service provision, and capacity strain across social service and healthcare settings. In both settings, there are similar capacity constraints and strategies to manage capacity such as culturally tailoring services to more efficiently and effectively meet client needs, spreading awareness through word-of-mouth to promote care-seeking, and expanding access and accessibility through building trust in existing services and referral to other organizations. Capacity is reinforced by adaptation, including tailoring delivery based on available human capacity and capabilities, and culturally and linguistically adapting services based on knowledge of community context to ensure accessibility and impact.

In addition to capacity constraints within their respective settings, participants elaborated that coordination between social service providers and clinical settings is in and of itself a strain on capacity. For example, when the health system is strained there may be less support available to ensure screener completion; or work to coordinate ambiguous referrals can strain the social service system. Participatory activities helped clarify a shared vision for bidirectional referral between health and social service providers. Further, the map helped nuance the operationalization of bidirectional referral by highlighting the need to build capacity for coordination activities and underscored the importance of complementing any new technology fixes with scaffolded organizational and community support. Phase 1 insights raised questions about the impact of capacity on screening and referral completion and how community members experience the process that helped inform exploration in subsequent phases.

#### 2.3.3 Phase 2 outputs

**Figure 2:**
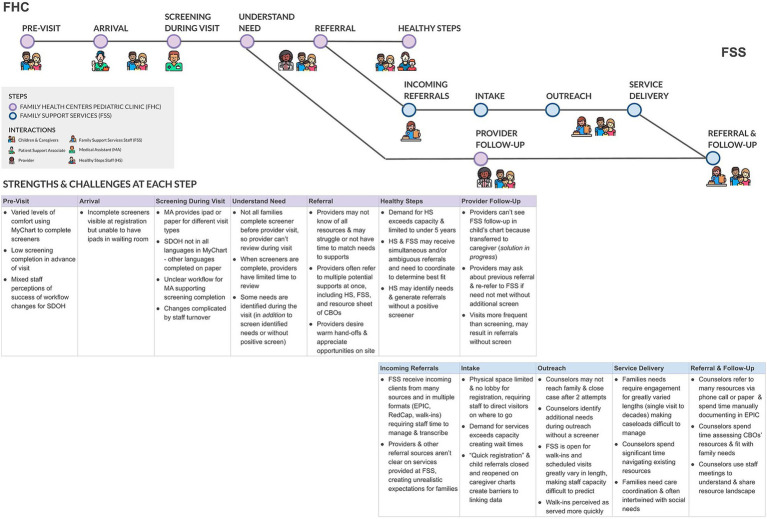
Process map depicting key steps in the screening and referral process, interactions, strengths and challenges.

**Figure 3:**
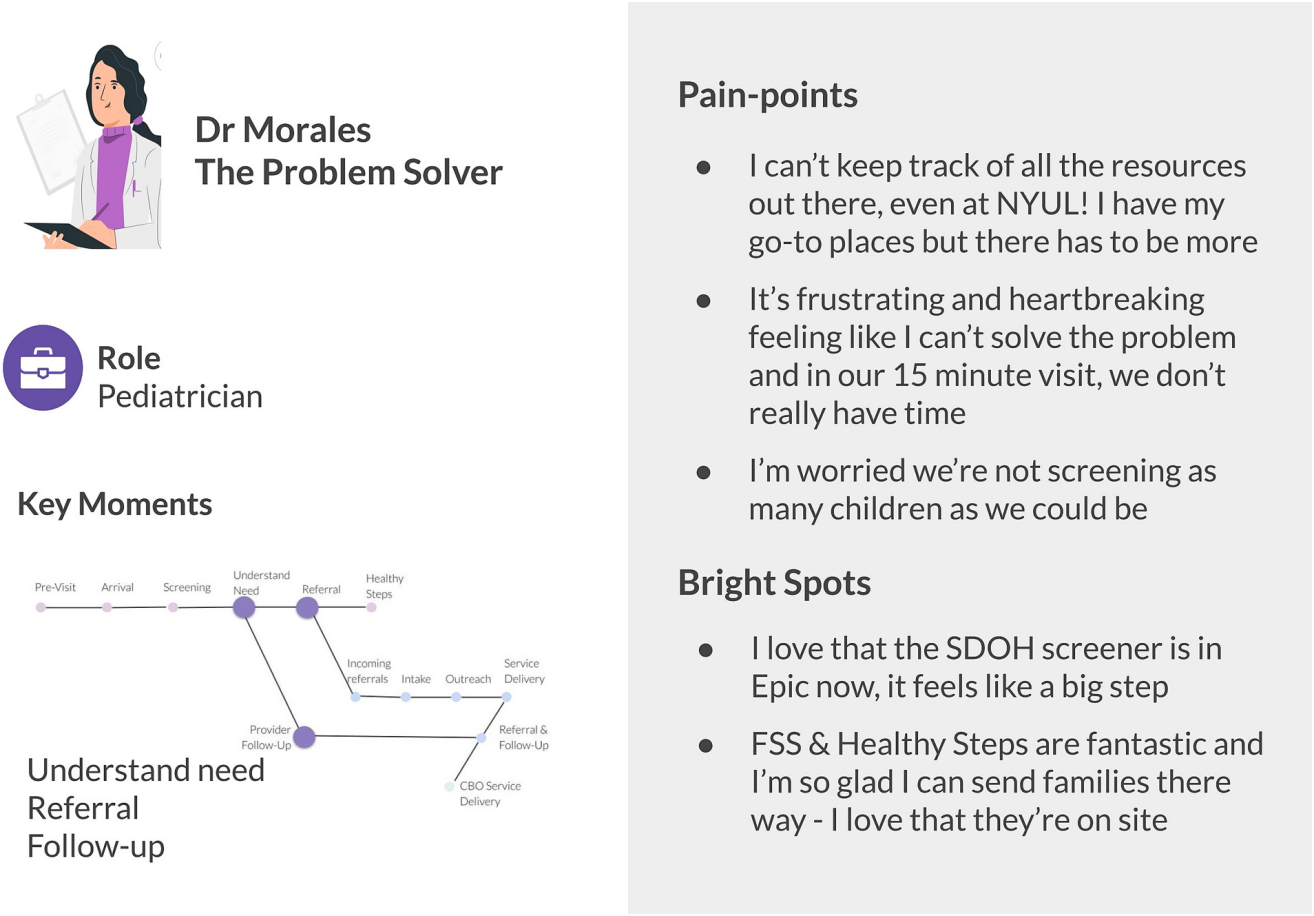
Illustrative persona overview including role, key moments in the screening and referral process, and pain-points and bright spots. The persona image illustration is by Storyset.

In Phase 2, outputs of design thinking activities were captured in a series of experience overview visuals describing activities throughout the screening and referral process and strengths and challenges at each step (See Figure 2). The process crossed institutional boundaries and included process overviews for the practice, social service providers within the FHC, and CBOs within the broader CCL. Versions of the process maps also included documentation of key interactions between stakeholders and use of supporting technology at each step. Persona overviews (See Figure 3) included roles, key moments for that persona in the broader experience, and “pain-points” and “bright spots” across the experience. Common terminology in design thinking, pain-points describe points of friction, frustration, or issues in an experience and bright spots describe highlights or elements of the process that work well from the persona’s perspective.

#### 2.3.4 Phase 2 insights

Visuals helped operationalize the process of addressing SDoH by complementing existing workflow documentation with additional activities that occur regularly in real world practices, including activity outside of institutional boundaries. Further, results in Phase 2 uncovered how individuals experience the structural dynamics identified in Phase 1. For example, a key insight from Phase 1 was the parallels in capacity dynamics in social service and clinical settings and Phase 2 results helped articulate how capacity strain manifests at the individual level. In the clinical setting providers described experiencing capacity strain as limiting their time in clinical encounters. If they saw a positive social need screen or a need came up in conversation during the encounter, making a referral to all possible options saved the provider time navigating a difficult to understand resource landscape and helped them feel someone would be able to provide support. In social service settings, providers described receiving referrals that needed to be re-referred to a different CBO to more appropriately address the identified need.

This work also challenged initial assumptions based on performance metrics that low screener completion was a key bottleneck. It uncovered that increasing screener completion may shift bottlenecks and strain capacity downstream in the system, such as straining community social service providers. Building on the example mentioned above, rapidly increasing screener completion could lead to an increase in multiple simultaneous referral that requires additional coordination by CBOs to re-refer or re-assess reason for referral, straining social service providers. Therefore, a key insight of Phase 2 was that the lack of clarity around current organizational services and processes for managing referrals could worsen existing capacity strain, which could be exacerbated without careful implementation of technological solutions that enable rapid increase in screening and referral. Phase 2 insights underscored the importance of role clarity and building coordination capacity and raised questions about how capacity bottlenecks impact key metrics, like patient throughput, and other difficult to measure metrics, such as drop-offs after referral or changes in rates of care-seeking.

#### 2.3.5 Phase 3 outputs

**Figure 4:**
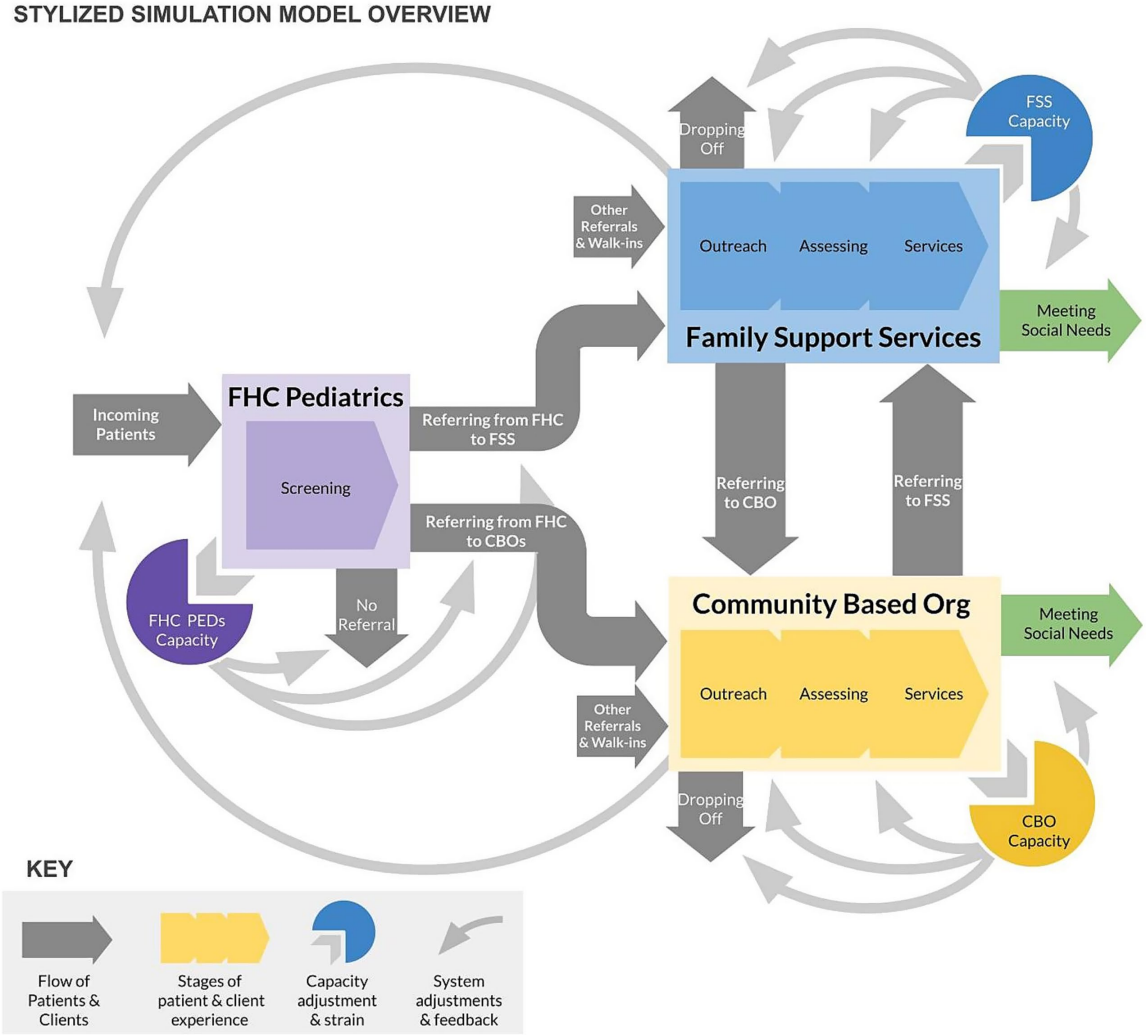
Schematic overview of the system dynamics simulation model depicting patient flows and capacity feedback dynamics.

**Figure 5:**
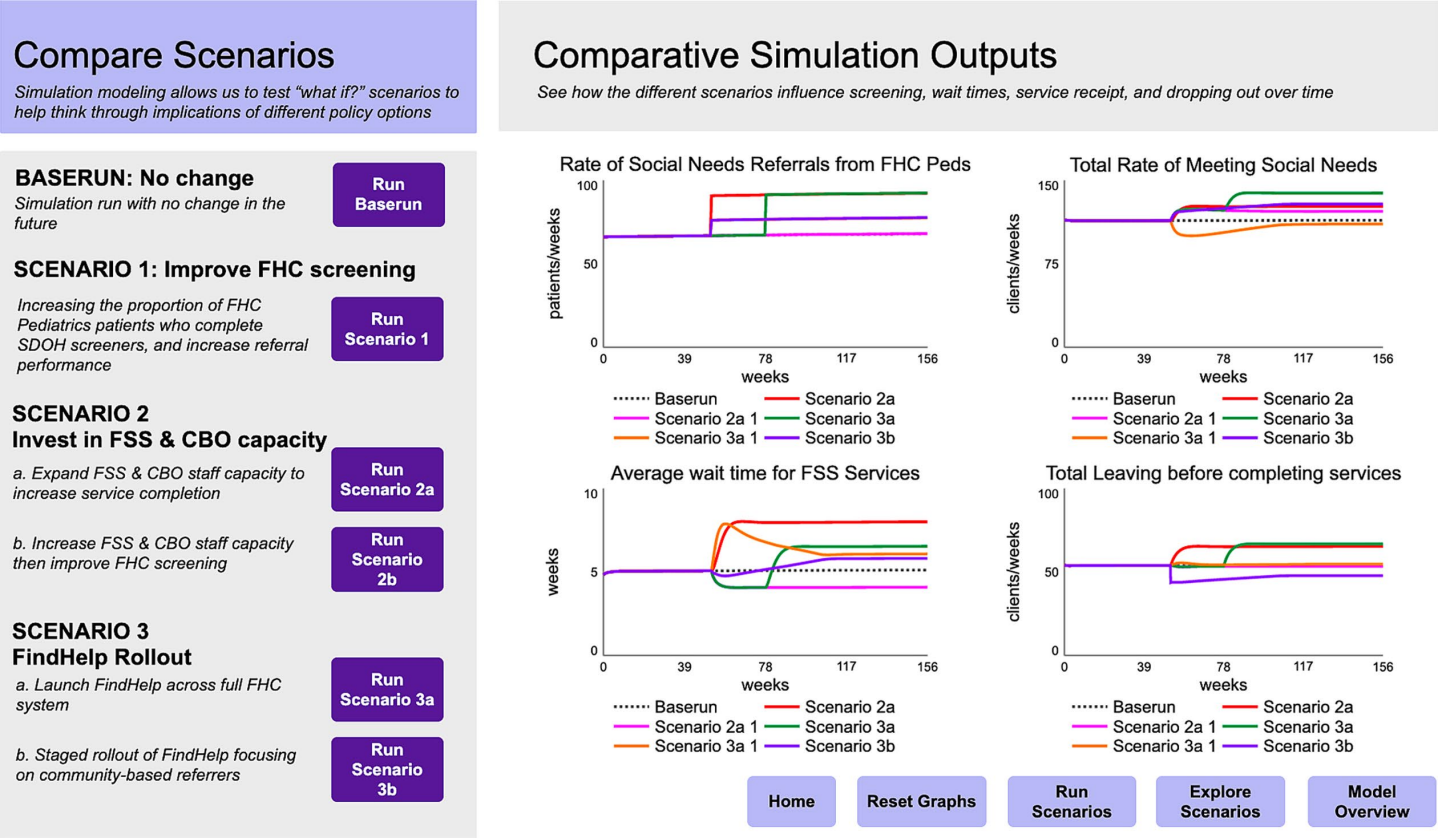
Demonstration of simulation model interface comparing scenarios of system transformation.

System dynamics modeling in Phase 3 produced a strategic simulation model that represented an abstracted version of the healthcare SDoH screening and referral system that focused on simulating the flow of patients within and between the selected practice in Phase 2, social service providers within the health system, and a generic external CBO (see Figure 4 for a schematic overview of the model). The model was calibrated using FHC data on patient visits, referral rates to internal and external social service providers, wait times to see social service providers, onward referral rates, and rates of patient loss to follow-up at each stage of the journey. The model was then used to create an interactive interface to allow users to project impacts of interventions on multiple outcome indicators over time (See Figure 5). The interface focused on key scenarios identified by the core team in consultation with institutional leadership, including:

1. Improvements to FHC screening rates.
2. Expand internal social service & external CBO staff capacity to increase service completion.
3. Sequenced increase to internal social service & CBO staff capacity followed by improvements to FHC screening.
4. Full launch of a technology solution to support screening and referral across FHC.
5. Staged rollout of a technology solution, prioritizing community-based referrers.

#### 2.3.6 Phase 3 illustrative insights

Simulation scenario analysis allowed the team to test the logic of emerging recommendations through mathematical simulation grounded in health system data and insights from prior stages. The analysis showed that increasing healthcare screening and referral may strain capacity at internal social service providers and at CBOs receiving referrals, and therefore limit their ability to actually meet needs and significantly increase wait times and potential patient loss to follow-up. A staged strategy to increase receiving capacity at internal and external providers can create an environment in which future improvements to screening and referral rates can better keep up with demand for services. Finally, system-wide rollout of network referral through a technology solution risks combining additional demand at referral agencies with reduced capacity as facilities adapt to new technology and administrative burdens, resulting in wait times and decrease in service provision overall. An alternative strategy that invests in streamlining and prioritizing referrals through the use of CHWs, for instance, may help to mitigate additional strain from ambiguous or duplicate referrals and smooth rollout.

#### 2.3.7 Resulting recommendations

The three phases resulted in a set of recommendations in four areas: (1) SDoH screener completion, (2) Managing social needs referrals, (3) Receiving referrals and downstream processes, and (4) Inflows to care. For SDoH screening, the team recommended a staged approach to increase screening completion that addressed downstream processes first, followed by phased support for screener completion. New clinical workflows can enable multiple options for screener completion to create efficiencies through patient self-service while creating mechanisms of support for patients that need it.

The team recommended more effectively managing referrals by clarifying roles to define who is supporting what task and further differentiating tasks between roles, including defining primary users of technological solutions to manage referrals, to reduce capacity strain. Further, recommendations included guidance around the use of CHWs. While calls for additional CHWs were not new, the team recommended a more nuanced deployment. The team recommended developing an expanded CHW workforce to be deployed in practices with oversight from a central department to enable supported screener completion, help ensure referral accuracy, and build coordination capacity with internal social service providers on referral follow up. Finally, the team recommended that the health system use a hybrid approach to managing referrals where CHWs and internal social service providers are embedded in clinical settings to enable integration with care teams and create conditions for tiered care coordination based on complexity of needs.

For receiving referrals, insights suggest investing in internal social service provider capacity and co-designing internal workflows to enable the hybrid approach to managing referrals described above. Outside of the health system, the team recommended collaboratively defining cross-entity responsibilities and setting expectations across CBOs. Investing in CBOs and exploring the possibility of embedded health system support also may help reduce risk of capacity strain and build capacity for coordination. Exploring new inflows to care, such as leveraging school-based health centers, could help strengthen access to healthcare and overall relationships with families in the community essential to CCLs. Ongoing evaluation of inflows to care will help the CCL investigate the impact of strengthened screening and referral pathways on subsequent care seeking, and eventual health and social outcomes.

## 3 Discussion

This case study demonstrates the value of combining community-based system dynamics and design thinking to understand the multi-level complexity of a public health challenge and propose feasible solutions. Integrating these methods generates insights at multiple levels, including connecting holistic system understanding to individual experiences of system structure. While calls for the combination of these methods are not new, there are limited examples in practice. Further, most calls in the literature point to the promise of a potential sequence in which systems approaches are used to understand a problem and identify potential leverage points, and design methods are applied to develop contextually relevant solutions. This case illustrates the potential value of a more nuanced integration of methods.

In this section, we discuss the value of methodological innovation for intervention planning, strategic implementation, and sustaining change. We close by reflecting on the specific application described in this case as a lens to identify unique assets that enabled methods integration.

### 3.1 Intervention planning: participation and systemic understanding

SDoH are inherently dynamic, likely to change over time and vary by individual preferences and circumstances. A community’s needs for social services, resources to address them, and relationships to secure them are all embedded in system structure. Therefore, interventions to address SDoH require understanding system structure and sharing diverse perspectives of those embedded in systems to collaboratively plan interventions. Community-based system dynamics and system dynamics simulation can enable practitioners to understand, situate, and plan interventions in their structural context (43, 51). Further, participation underpins community-based system dynamics and design thinking approaches to both understand systems and co-create change grounded in people’s unique perspectives of the system (43, 56). When combined, these methods support intervention planning that conceptualizes the intervention as dynamic, grounds the change in real world contexts, and is co-created through participation.

In this case, participatory group model building activities in Phase 1 created space to share diverse perspectives and understand how addressing SDoH happens in the real world rather than ideal or controlled settings. Participants thus gained an initial sense of types of realistic interventions and helped foster a partnership where both healthcare providers and CBOs saw strategic advantage in bi-directional learning to better meet community needs. In Phase 2, building on this foundation, design thinking tied structural understanding to individual experiences of a variety of stakeholders, from patients to medical assistants to social service providers. The unique value of group model building informing design activities enabled the core project team to ask the right contextual, structural questions to deeply understand peoples’ lived experiences and derive key insights around the necessary supportive infrastructure to facilitate success. In Phase 3, system dynamics simulation demonstrated how interventions could work in the system grounded in participants’ insights. Further, the interactive interface put insights into the hands of key stakeholders as part of intervention planning to generate buy-in for recommended changes.

Further, the order of methods applied in this case challenged the common assumption that systems science should be used first for problem understanding, followed by design thinking for solution development. This case illustrates the value of system dynamics for solutioning and design thinking for problem understanding in intervention planning, such as using design activities to understand individual experiences of systems and system dynamics modeling for solution development to simulate the effects of multiple potential scenarios.

### 3.2 Strategic implementation: operationalizing change

In addition to the intervention itself, methodological integration can support strategic implementation of interventions to address SDoH and improve public health. System dynamics emphasizes operational thinking as a key tenet to enable creating real world change (40). Design thinking is a solution-oriented approach, grounded in iterating on interventions in part to refine how a solution is operationalized to improve its impact (57). Together, methods integration provides tools to operationalize assumptions about implementation and translate systemic insight into tangible actions that support decision-making. This includes sequencing activities to think strategically about stages of implementation and their potential impacts.

For example, in this project, operationalizing implementation via a system dynamics simulation model led to more nuanced insights around sequencing support for increasing screening and managing referral to avoid shifting bottlenecks downstream. Increasing screening without subsequently building capacity among referral receivers risks increasing wait-times or losing more patients to follow-up. Operationalizing implementation using a simulation model helped inform a sequence of activity starting with building capacity of social service providers downstream, followed by staged support for screener completion through the use of CHWs and creating efficiencies through patient self-service.

Operational thinking can also help with closer coupling of planning and evaluation to inform implementation. If planning relies solely on existing evaluation metrics, it risks limiting change efforts to interventions that show impact on existing metrics. Operationalizing implementation through simulation can help identify metrics that might not be evaluated, but may be an indicator of success that’s difficult to measure or outside institutional bounds. In this case, screener completion is a key institutional metric. Optimizing implementation to solely increase screener completion could create bottlenecks downstream that undermines the reason for screening, subsequent referral and addressing SDoH. Operationalizing implementation helped move implementation beyond existing metrics and helped inform future evaluation by identifying new potential balancing metrics, such as patients lost to follow-up, that could be an otherwise hidden effect outside of institutional bounds.

### 3.3 Sustaining change: engaging community

Addressing SDoH requires systemic change and sustained effort over time, and therefore must be supported by deep community engagement. Community-based system dynamics is grounded in the belief that communities are at the center of both systemic understanding and driving systems change. This stands in contrast to other approaches in systems science that use participation functionally to develop more accurate models or solely use simulation to develop systems insights. In moving beyond insight to lasting change, community-based system dynamics emphasizes participation and building capabilities as core components of the approach (43). In parallel, human-centered design evolved into design thinking in part as a way to democratize design tools and put them directly into the hands of community members to facilitate sustained change (58, 59). Similarly, this is one way design thinking can be differentiated from other forms of design, such as user experience design (60), that may have elements of participation but not deep community engagement. When combined, community-based system dynamics and design thinking show promise for moving beyond use by experts to application by people embedded in the system to support sustaining change.

In this case, leveraging a core project team helped support future replication by seeding team capabilities, developing materials, and critically reflecting on the methods’ utility. Further, during the project period there was immense value in having activities facilitated and synthesized by an internal team rather than solely external collaborators or consultants. The team had unique insights into the broader institutional and historical context that potentially resulted in a more sustainable solution. The intentional process of team formation helped build and strengthen relationships across departments, relationships that could serve as a foundation to sustain collaboration in the future.

Beyond the core project team, participatory activities created new avenues for community voice within and across institutional boundaries. This included collaboration within teams, across departments, and across institutions. Further, engaging in culturally diverse communities, such as Sunset Park, demands a culturally competent framework that acknowledges the lived experiences of community members, particularly those from historically marginalized groups. Many CBOs’ existing practices offered invaluable insights into how health-related topics can be framed and delivered in ways that resonate with diverse community members. This bi-directional learning was a cornerstone of the approach, emphasizing that healthcare systems not only impart resources and frameworks to CBOs but also adapt and learn from the culturally nuanced strategies already effectively implemented by these community partners. In this study, culturally responsive strategies were implemented throughout the CCL design process, including incorporating trusted cultural liaisons and adapting participatory methods to align with community norms. This approach allowed the project to bridge holistic system insights with the everyday lived realities of community members, enhancing both the relevance and potential impact of the intervention.

Foundationally, group model building lifted voices of varied perspectives and as described, informed subsequent design activities to understand peoples’ intimate everyday experiences. Beyond supporting intervention planning, deep understanding helped build structural empathy for people’s lived experiences, whether that be at work or in care-seeking. This helped strengthen relationships and can hopefully serve as the foundation for sustained change.

### 3.4 Reflecting on assets enabling methods integration

This project featured a unique set of assets and contextual factors that likely played a role in the impact of successful methodological innovation. The broader initiative to create and strengthen a CCL in Sunset Park was funded by a significant philanthropic gift. This provided resources for activities and also created pressure to show results and move rapidly. While significant resources may not be a requirement for methodological innovation, resources help demonstrate commitment to change and support action.

Program leadership, including one of the program leads and the external consultants, have significant experience with the methods that allowed for innovation. Advanced skills helped enable adaptation and experience with many variations of methods applications to drive creativity and flexibility in the approach. The project also had strong institutional buy-in and support. Institutional leadership was regularly engaged, including providing feedback on methods and requesting regular updates on emerging insights and recommendations. Future efforts to integrate methods should intentionally cultivate institutional leadership buy-in through ongoing engagement. Project leadership valued developing team capabilities internally, rather than outsourcing, that led to intentional team formation. Having a large, embedded team and explicitly building that team’s capabilities and connection to one another was a unique and essential asset. The team included functional diversity, such as members from program leadership and staff, social services, clinical partners, and community liaisons. Further, a trusted working relationship among the core project team enabled experimenting with methods without risk of perfectionism or failure. Future methods innovation should strive for intentional team formation and capability development.

The project capitalized on a fortuitous moment in time. The FHC has a long history of community engagement and therefore a strong foundation of community connections. Recent Medicaid policy change on SDoH screening and referral catalyzed momentum for action across the health system and CBO partners. While not all methodological innovation must wait for the perfect moment, situating efforts in historical context and broader efforts for change may help practitioners identify opportunities to leverage momentum. Finally, the project committed to systems change as the end goal, rather than rigorous methods application. While there is value in highly controlled methodological application, innovation with a clear vision of creating change requires flexibility and prioritizing adaptation when needed to ensure action.

## 4 Conclusions, limitations, and future directions

Systems science and design thinking methods integration shows promise to effectively develop strategies and solutions to address SDoH and improve public health. This case study represents one application of combining community-based system dynamics and design thinking. Integrating methods generates insight at multiple levels, including connecting holistic system understanding to individual experiences of system structure and operationalizing and translating insights into action. Future research should continue to develop the methods and explore deeper and/or iterative integration between methods.

Limitations of this contribution are that it is a case study describing a process at a single site, potentially limiting generalizability. Existing advanced team capabilities enabled the methodological development and integration described here, which may not be present among other sites or groups. However, the methods applied are not novel and have been described and replicated elsewhere. In addition, the proposed value of methodological innovation for intervention planning, strategic implementation, and sustaining change is based on observation. Additional applications can help document and build evidence of the observed value of this integration, or identify other potential utility. Future efforts should include cross-training between systems science and design thinking methods and documenting team formation and capability development activities to support replicability of this approach or adaptation to different project contexts and team configurations.

This case study underscores that SDoH and the strategies to address them are inherently products of systems. And systems are produced, perpetuated, and improved by the people living, working, and making decisions within them. Therefore systems are essential subjects of study in and of themselves, but this work requires moving beyond studying systems to innovating on methods to produce new models of evidence, new ways of working to better translate insight to action, and action to community impact. Further, systems are designed. We cannot stop at studying and describing systems. We need methodological innovation to interrogate who systems are designed for and designed by and to empower communities to transform systems.

## Data Availability

The raw data supporting the conclusions of this article will be made available by the authors, without undue reservation.
